# Squishing sound heard following an intra-articular shoulder injection with fluid and air is associated with higher efficacy: A retrospective analysis

**DOI:** 10.3233/BMR-210360

**Published:** 2023-03-15

**Authors:** Jan M.A. Mens, Ronald T.M. van Kalmthout

**Affiliations:** a Department of General Practice, Erasmus University Medical Centre, Rotterdam, The Netherlands; b MSK Clinic, Leiden, The Netherlands

**Keywords:** Capsulitis shoulder, intra-articular injections, glucocorticoid, efficacy, accuracy

## Abstract

**BACKGROUND:**

Accuracy of blind intra-articular injections for the shoulder is rather low. It is unclear whether accurate injections for capsulitis of the shoulder are more effective than inaccurate injections.

**OBJECTIVE:**

It has been hypothesized that a squishing sound following an intra-articular injection with a mixture of air and fluid means that the injection was accurately placed and that the efficacy of accurately placed injections is greater than that of inaccurate injections. The aim of the present study was to test the hypothesis that a squishing sound following an injection predicts a better clinical result.

**METHODS:**

Files were selected of patients with capsulitis of the shoulder, who were treated with an intra-articular injection containing a mixture of triamcinolone, lidocaine, and air. After the injection, the shoulder was moved to determine whether a squishing sound could be produced. Efficacy was measured after two weeks according to the Patient Global Impression of Change scale. Differences in efficacy between injections with and without a squishing sound were expressed as an odds ratio.

**RESULTS:**

Sixty-one patients were selected. Squishing was heard after 47 injections (77%). Two weeks after the injection, a positive outcome was reported by 49 patients (80%). When squishing was heard, the effect was positive in 42 of the 47 patients (89%) and when no squishing was heard, the effect was positive in 7 of the 14 patients (50%). The odds ratio was 8.4 (95% CI 2.1–34.0; p= 0.003).

**CONCLUSION:**

Efficacy of injections with a mixture of triamcinolone, lidocaine, and air for capsulitis of the shoulder is significantly greater when a squishing sound was heard after the injection. We hypothesize that squishing is related to accuracy and accuracy to efficacy. A future study with X-ray arthrography is needed to verify both hypotheses.

## 1. Introduction

Capsulitis of the shoulder is a common condition in middle-aged people. Capsulitis is characterized by pain, especially at night, and limitation of shoulder movement in all directions [1]. Risk factors for developing capsulitis of the shoulder are: higher age, female sex, and diabetes [2].

The natural course of capsulitis has been described as evolving through three phases: pain, stiffness, and recovery [3]. According to old studies the duration of the first phase ranges from 10–36 weeks, the stiffness phase from 4–12 months, and the recovery phase from 5–26 months and the entire course 12–48 months [3]. However, recent studies show that full recovery may take multiple years [3].

Most studies recommend intra-articular injections for treatment of pain, and exercise for treatment of stiffness [4, 5, 6]. A recent overview summarized eight meta-analyses of randomized controlled trials regarding conservative treatment options for capsulitis of the shoulder [7]. The authors concluded that an intra-articular injection with a corticosteroid confers greater pain relief in the short-term (0–12 weeks) than a placebo injection.

However, accuracy of blind intra-articular injections for the shoulder joint is often rather low [8]. In a systematic review, it was shown that in about half the selected studies the score was lower than 90% and sometimes far below 90%. In one study, the accuracy was only 26.8% [9].

In all those studies contrast dye was added to the injected fluid, and X-rays or MRI were used in order to determine accuracy. In 1991, Jacobs et al. suggested another method for determination of accuracy [10]. They wrote: “… intra-articular injection of a small volume of steroid into the shoulder may be less reliable than when a larger volume containing air is injected. Air in the shoulder results in a ‘squelch’ when the joint is subsequently moved.” According to these authors, the squishing sound following injection of a mixture of air and fluid indicates that the injection has been given correctly.

After reading the article by Jacobs et al., we always add 2 ml of air to the fluid when injecting corticosteroids into the shoulder joint. Since then, we also make a note in the patient’s file indicating whether or not a squishing sound was heard. The aim of the present study was to test the hypothesis that squishing after an injection predicts a better clinical result. More specifically the aim was to investigate the relation between squishing (yes/no) and the Patient Global Impression of Change two weeks after the injection.

## 2. Materials and methods

### 2.1 Design

We retrospectively analyzed the files of patients with phase-1 capsulitis of the shoulder who were treated with an intra-articular injection in the shoulder in our clinic between July 1, 2010 and July 1, 2016. This study was approved by the Institutional Review Board (IRB) of Erasmus University Rotterdam, MEC-2019-0138. The IRB concluded that written informed consent was not needed since the study was retrospective and the data were collected and analyzed anonymously.

### 2.2 Selection of files

Inclusion of patients was based on medical history, clinical examination, and ultrasound investigation. If indicated, an X-ray was taken. Data were collected from patients meeting all of the following criteria: 1) pain in the acromial region (with or without radiation into the arm); 2) restriction of passive anteflexion, and active internal rotation; 3) at least a 20-degree restriction of passive external rotation and of glenohumeral abduction; 4) pain at passive anteflexion as well as external and internal rotation; 5) no clinical signs of subacromial pain syndrome (SAPS) or osteoarthritis.

We excluded patients who had a history of fracture of the humerus and/or scapula, patients with a rheumatic disease, bilateral shoulder pain, and those who had had an intra-articular injection into the studied shoulder within the past 3 months. Patients with full-thickness rotator-cuff rupture and/or peri-articular calcifications larger than 3 mm were also excluded.

Sample size calculation with alpha 0.05 and beta 0.80 showed that the number of needed dossiers was 88. This value was based upon the estimated proportion of patients with squishing was 67%, efficacy was 85% in case of squishing and 50% in those without. Moreover, with the assumption that 25% of the dossiers had to be excluded.

### 2.3 Protocol

The shoulder joint was treated with a blind injection using the method described by Cyriax [11]: “… the patient sitting and the shoulder internally rotated. The needle punctured the skin 2 cm below the point where the lateral edge of the acromion and the lower edge of spine of the scapula meet. Then the needle was moved in the direction of the point of the coracoid process until the humeral head was felt with the needle.” Some resistance is felt as the capsule is pierced, and the patient usually complains of pain at that point [10].

A mixture containing 0.5 ml triamcinolone 40 mg/ml, 3.5 ml lidocaine 1%, and 2 ml air was then injected. A 50 mm 22G needle was used. After the injection and removal of the needle, the physician rotated the slightly abducted arm internally and externally a few times to check for a squishing sound.

The efficacy of the injection was measured after 2 weeks by asking the patients to give their impression on a six-point Patient Global Impression of Change (PGIC), which was documented in their files. A response of ‘much better’ or ‘completely recovered’ was defined as positive; ‘somewhat better,’ ‘unchanged,’ ‘somewhat worse’ and ‘much worse’ as negative. Some patients scored their improvement by giving a percentage. In such cases, 50% improvement or higher was scored as positive and a score < 50% as negative.

In addition to the extent of restriction of range of motion (in degrees) and squishing (present or absent) data were collected regarding factors that might influence the success of the intervention: age, sex, diabetes (present or absent), and duration of symptoms in months.

### 2.4 Statistical analysis 

Normally distributed continuous variables are presented as mean and standard deviation; non-normally distributed continuous variables as median and interquartile range. Differences between responders and non-responders are analyzed with an independent samples t-test for normally distributed continuous variables (age), with a Mann-Whitney test for non-normal distributions (duration of complaints, restriction of external rotation and abduction) and with a chi-square test for categorical variables (gender and diabetes). Differences in efficacy between injections with and without a squishing sound are expressed as odds ratios computed in a univariate logistic regression with the clinical effect (positive or negative) as the dependent variable. Odds ratios are presented with a 95% confidence interval (95% CI). Analyses were performed using SPSS version 27. P< 0.05 is considered statistically significant.

## 3. Results

### 3.1 Squishing

**Figure 1.**
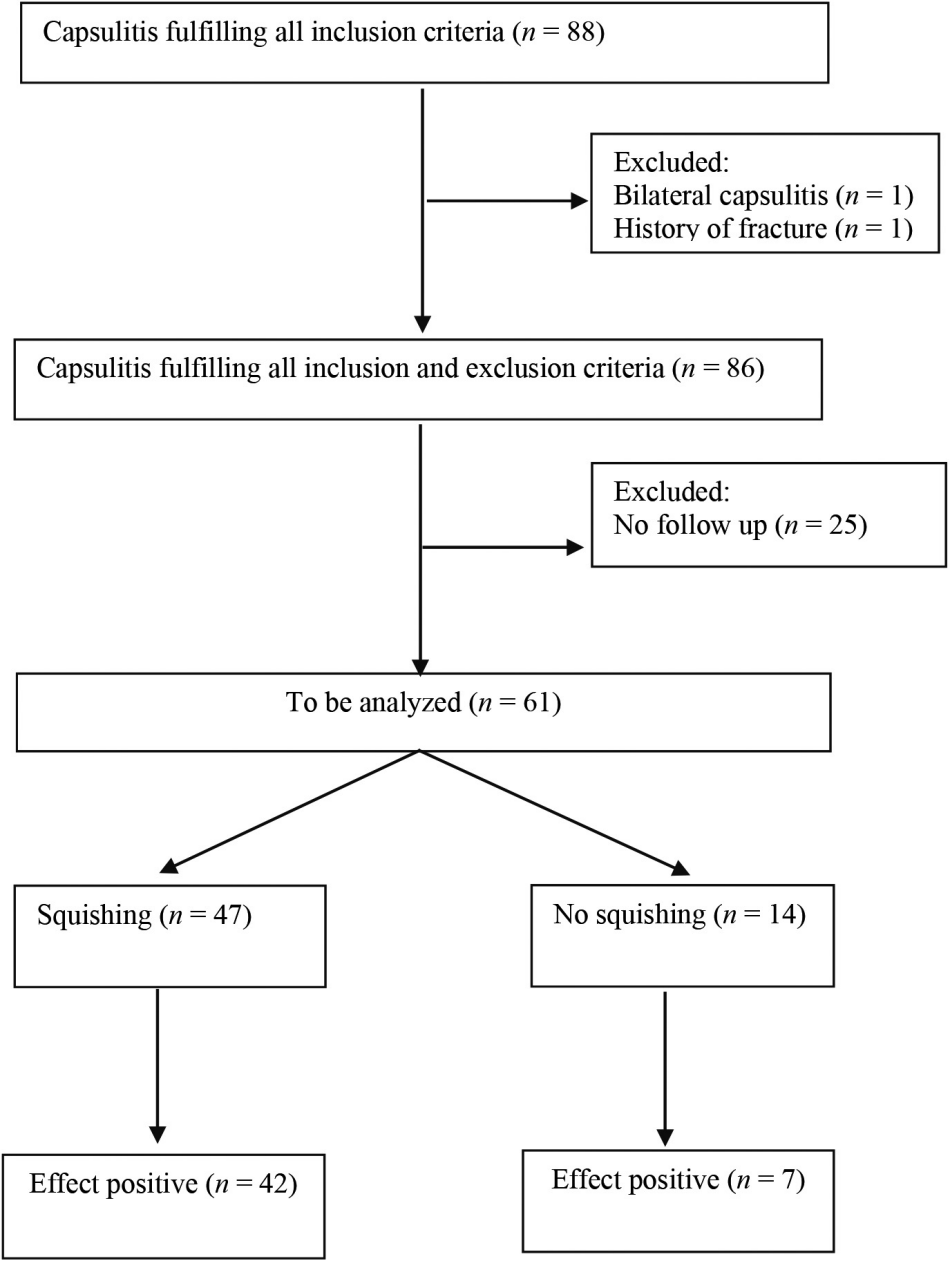
Flow diagram.

In the 6-year period, 88 patients fulfilled all inclusion criteria (Fig. 1 and Table 1). Two patients were excluded. Efficacy data were not recorded in 25 dossiers. Mostly because patients cancelled the appointment for follow-up. Hence, 61 cases were available for analysis. A squishing sound was heard following 47 of these 61 injections (77%).

### 3.2 Efficacy

Two weeks after the injection, none of the 61 patients reported worsening of their symptoms. A positive effect was reported by 49 patients (80%) and a negative effect by 12 patients (20%).

**Table 1:** Characteristics of the 86 included patients

Variable	Responders n= 61	Non-responders n= 25
Age in years, mean (standard deviation)	60 (10)	54 (6)${}^{\text{a}}$
Gender, percentage women	51	56
Diabetes, percentage	8	4
Duration of complaints in months, median (IQR)	5 (6)	4 (3.8)
Restriction of external rotation in degrees, median (IQR)	40 (20)	40 (20)
Restriction of abduction in degrees, median (IQR)	40 (15)	40 (23)
Squishing (%)	77	80

IQR = Interquartile range. ${}^{\text{a}}$Differences between groups significant p= 0.006.

### 3.3 Squishing and efficacy

When a squishing sound was heard, the effect was positive in 42 of the 47 patients (89%) and when no squishing sound was heard, the effect was positive in 7 of the 14 patients (50%). A comparison of these outcomes showed an odds ratio of 8.4 (95% CI 2.1–34.0; p= 0.003) in the univariate analysis with Nagelkerke pseudo $R^{2}=$ 0.22.

### 3.4 Check for selection bias due to missing data

Twenty-five cases were lost to follow up. Besides the fact that the non-responders were on average 6 years younger than the responders, no significant differences were found between responders and non-responders (Table 1).

## 4. Discussion

The present study showed that patient scores on the PGIC scale reported two weeks after a corticosteroid injection were strongly related to squishing. Selection bias due to missing data seemed unlikely. 

To explain this strong association between squishing and efficacy, we hypothesize that squishing is related to accuracy and accuracy to efficacy.

### 4.1 Squishing and accuracy 

It is our hypothesis that squishing is related to accuracy. Two previous publications used the squishing sound as evidence that the injected mixture had reached the joint cavity of the shoulder [10, 12]. The association between squishing and accuracy has never been proven for the shoulder, as far as we know. The following facts support our hypothesis:

A study in the knee showed that after injection of a mixture of local anesthetic, corticosteroid, contrast dye, and 1 to 2 cc of air, a squishing sound was heard after 85% of intra-articular injections and in none after injections placed extra-articularly [13]. Glattes e.a. hypothesized that the noise in the knee “… is produced as the air passes from the femoral-tibial compartment to the suprapatellar cavity.” [13] We theorize that the noise in the shoulder arises when the air will be moved during rotations from one part of the joint cavity to another part.

Sounds following injection of a mixture of air and fluid into a joint after double-contrast arthrography are well-known. The American College of Radiology writes on their patient-information site that after arthrography “… the patient may … hear gurgling when the joint is moved” [14], and, as far as we know, squishing sounds have never been heard after intra-articular injections with fluid only. In case of inaccuracy or leakage of blind posterior injections, the fluid appears almost always in the infraspinatus and/or teres minor muscle belly [15, 16, 17, 18]. It is theoretically unlikely that an injection of a mixture of fluid and air in the belly of those muscles could result in a squishing sound when the shoulder is moved by the physician.

### 4.2 Accuracy and efficacy

It is our hypothesis that accuracy is related to efficacy. That inaccuracy reduces efficacy seems obvious, but a firm association between accuracy and efficacy was, as far as we know, not been assessed in previous studies [19, 20, 21, 22]. In two of those studies, a strong conclusion could not be drawn because in the analysis, the results of the patients with capsulitis were pooled with those of patients with various other diagnoses [19, 20]. In two other studies, the efficacy of accurate injections was not significantly greater than that of inaccurate injections [21, 22]. These results could have been due to the rather small number of inaccurate injections (seven and eleven). Another explanation for the weak association between accuracy and efficacy in the aforementioned studies could be that leakage of fluid outside the joint was not considered as a possible explanation for inefficacy. Leakage after injection is quite common. A systematic review comprising 21 publications mentioned the appearance of extra-articular contrast in 1.0%–51.0% of the cases [8]. Rutten et al. injected 50 shoulders guided by ultrasound and 50 by fluoroscopy, reporting a minimal amount of extra-articular contrast in 24% of all procedures, but a massive amount in 27% of them [23].

In most studies on accuracy, the appearance of contrast fluid in the joint was defined as a successful injection. However, this definition is debatable. Rutten et al. showed that even significant leakage of contrast did not compromise the diagnostic quality of the arthrogram [23]. However, significant leakage may reduce efficacy. It is theoretically possible that the production of a squishing sound after injection could mean that a large part of the fluid has reached the joint cavity without significant leakage. This could be a better indicator for correct placement than sufficiency of fluid reaching the joint, for purposes of producing an acceptable arthrogram.

The present study has much in common with a former study comparing the effect of ultrasonography guided and blindly given intra-articular injections [25]. In that study ultrasonographic given injections were more effective to reduce pain and to improve range of motion and function two weeks after the injections. The authors suggest that the injection technique in their study was related to accuracy and accuracy to efficacy, but a check for accuracy was missing.

### 4.3 Limitations

One limitation of the study is that the patients were not ‘blinded’ for the squishing sound. Theoretically they could have been more prone to report a positive result when they heard the squishing sound.

Another limitation is that we used only one effect measure (PGIC) and only one evaluation. Most prospective studies use two or three measures to determine clinical changes. Although the use of only one effect measure is a limitation, patient’s opinion is very responsive and, at least in phase 1, seems to be more relevant for the patient than surrogate measures such as improvement of range of motion or strength [26, 27, 28].

Moreover, the retrospective design and the small amount of only 12 patients with a negative result are limitations. A blinded control group with an injection of 4 ml placebo-fluid with 2 ml air could have made the conclusions more robust. 

### 4.4 Recommendations 

The present study indicates that a prospective study should be performed, with a blinded observer for squishing and a blinded radiographic control for intra-articular air. It is a challenge to find a method to make the researcher more objective in the assessment of squishing.

## 5. Conclusion 

Hearing a squishing sound following an injection with a mixture of triamcinolone, lidocaine, and air correlates with a better short-term efficacy in patients with phase-1 capsulitis of the shoulder. It is quite probable that squishing is related to accuracy, and accuracy to a positive result. New studies are needed to assess the definitive causalities between squishing, accuracy and efficacy.

## Ethical approval

This study was approved by the Institutional Review Board (IRB) of Erasmus University Rotterdam, MEC-2019-0138.

## Funding

None to report.

## Informed consent

Not applicable.

## Author contributions

Both authors contributed to the formulating of the hypothesis. Both authors contributed to the collection of the data. JM was responsible for the statistical analysis, the raw writing of the manuscript and the correspondence. RK was responsible for the final version of the manuscript.
